# Study on Strain Characterization and Failure Location of Rock Fracture Process Using Distributed Optical Fiber under Uniaxial Compression

**DOI:** 10.3390/s20143853

**Published:** 2020-07-10

**Authors:** Shiang Xu, Shuangming Wang, Pingsong Zhang, Duoxing Yang, Binyang Sun

**Affiliations:** 1 State Key Laboratory of Mining Response and Disaster Prevention and Control in Deep Coal Mines, Anhui University of Science and Technology, Huainan 232001, China; shiangxu@aust.edu.cn; 2 School of Earth and Environment, Anhui University of Science and Technology, Huainan 232001, China; sxmtwsm@163.com (S.W.); binyangsun1993@163.com (B.S.); 3 Key Laboratory of Coal Resources Exploration and Comprehensive Utilization, Ministry of Land and Resources, Xi’an 170021, China; 4 Institute of Crustal Dynamics, China Earthquake Administration, Beijing 100085, China; yangdx@mail.iggcas.ac.cn

**Keywords:** uniaxial compression, rock fracture, distributed optical fiber, strain observation

## Abstract

A rock fracture test is a very important method in the study of rock mechanics. Based on the Mechanics Test System (MTS), the dynamic strain response of the failure process of cylindrical granite specimens under uniaxial compression was observed by using distributed optical fiber strain sensors. Two groups of tests were designed and studied for rock sample fracturing. The main comparison and analysis were made between the distributed optical fiber testing technology and the MTS testing system in terms of the circumferential strain response curve and the evolution characteristics of strain with time. The strain characterization of distributed optical fiber in the process of rock fracturing was obtained. The results show that the ring strains measured by the distributed optical fiber sensor and the circumferential strain gauge were consistent, with a minimum ring strain error of 1.27%. The relationship between the strain jump or gradient band of the distributed optical fiber and the crack space on the sample surface is clear, which can reasonably determine the time of crack initiation and propagation, point out the location of the rock failure area, and provide precursory information about rock fracture. The distributed optical fiber strain sensor can realize the linear and continuous measurement of rock mass deformation, which can provide some reference for the study of macro damage evolution and the fracture instability prediction of field engineering rock mass.

## 1. Introduction

Rock mass is a kind of complex geological body. The foundation of establishing the concept and theory of rock mechanics is to measure the parameters of rock mass [1]. The mechanical properties and failure mechanism of rock mass are affected by the mineral composition, cementation composition, formation environment, structure, and other factors. There will be great differences among different rock blocks [2]. Influenced by the surrounding environment, micro fissures will develop and continue to expand until they penetrate through the rock mass, which may further affect the strength and stability of the rock mass [3]. Furthermore, the anisotropy of rock usually makes these micro fissures randomly distributed [4,5]. At the same time, the uncertain parameters of rock composition make it irregular in the observation of fracture evolution. The research on the stress–strain characteristics and damage propagation of rock mass under load conditions is helpful for us to study the fracture and destabilization failure of rock materials and to further understand the mechanism of rock fracture evolution, which is of great scientific and engineering significance for carrying out better rock mass stability evaluation, fracture law analysis and quantitative analysis of rock mechanical characteristics.

Generally, many scientific researchers have carried out observations and tests on rock fracture evolution under uniaxial compression from different characterization parameters to discuss the cracking and propagation characteristics and laws of rock cracks [6,7]. The selection of the observation method is a key factor to accurately obtain rock mass parameters. Therefore, various new methods have been proposed in the field of rock mechanics. Acoustic emission (AE) technology has long been a widely used method for the fracturing test observation of rock mass [8]. Based on scanning electron microscopy (SEM), Zhao et al. observed the development process of the surface fracture during the fracturing process of a rock specimen, and studied the relationship between the density distribution and aggregate shape of the crack and the rock material and stress level [9,10]. Liu et al. studied the crack growth failure process of sandstone and granite under uniaxial compression by using real-time holographic interferometry, a high-resolution digital camera and computer image processing technology [11]. A computerized tomography (CT) machine is used for the real-time observation of the coal and rock failure process. The minor damage process of coal and rock mass under different loads is obtained through experiments, and the influence factors of the initial damage of rock are introduced [12,13]. Wei et al. and Fan et al. studied the feasibility of the application of fiber Bragg grating (FBG) surface sticking technology to the deformation monitoring of rock samples by using the methods of FBG and strain gauges [14,15]. Munoz H. et al. observed the strain characteristics of Pre-Peak and Post-Peak under uniaxial compression using three-dimensional digital image correlation (3D DIC) testing technology for sandstone [16,17]. Meanwhile, he also studied local damage and the progressive localization of porous sandstone during cyclic loading, and found out that DIC, as a non-contact test method, could provide records of the whole deformation process of the tested object [18,19]. Taheri A. et al. studied the influence of rock strength and confining pressure on the threshold value of cracks [20]. By setting 26 ultrasonic probes around the rock, He et al. have observed the velocity of an ultrasonic P wave during the fracturing process and studied the internal structure change during the process of rock deformation and instability [21]. Based on 3D digital image correlation technology, Ma et al. observed the whole failure process of the granite slab with a central circular hole under uniaxial compression and obtained the 3D displacement and strain of the observation surface during the failure process of porous rock [22]. Zhang et al. analyzed the uniaxial compression process of granite and the evolution characteristics of rock damage and failure by using the joint testing technology of acoustic emission and thermal infrared [23]. At present, there is still no reliable method to record and describe the surface stress–strain and crack development characteristics during the uniaxial compression of rock mass, and testing technology involving real-time and dynamic continuous observation is required. There are many difficulties in recording and recognizing the process of rock failure in rock deformation observation for underground engineering.

In recent years, the Brillouin optical time domain reflectometer (BOTDR) testing technology has been widely used in geotechnical engineering testing [24]. Compared with point measurement, it not only realizes linear distributed measurement but also has the advantages of anti-electromagnetic and noise interference [25]. Mendez A. et al. applied optical fiber to the detection of reinforced concrete structures and achieved good results [26]. Fuhr P.L. et al. embedded an optical fiber sensor in the bridge deck of an expressway to monitor the vibration response of the bridge, and the test results were in good agreement with the measured data using conventional methods [27]. Inaudi D. et al. used the Surveillance d’Ouvrages par Fibres Optiques (SOFO) system to test stability in civil engineering, for structures such as tunnels and dams [28]. Liu J. et al. used distributed optical fiber testing technology to monitor the deformation of a foundation pit [29]. Since the measurement is different for rocks and structures, there are not many studies on test method and device layout. Researchers pay more attention to the application of distributed optical fiber in the health monitoring of concrete structure or soil. At present, although there are some applications of distributed optical fiber in coal mine overburden deformation measurement and failure tests [30,31], the corresponding basic research is insufficient. As a result, the measurement advantage of distributed optical fiber testing technology makes it possible to test and realize the strain field by bending and continuously arranging it on the surface or inside the tested object. Therefore, it can be applied to the stress and strain monitoring of rock deformation and failure process [32,33].

In this study, Brillouin optical time domain reflectometer (BOTDR) testing technology was applied to realize the linear measurement of rock stress and strain. The optical fiber is wound and pasted on the surface of the rock sample in a circular direction, to obtain the circular strain on the length of the measuring line under the condition of uniaxial compression. By comparing the results of the optical fiber test and MTS test, the feasibility of distributed optical fiber is explored, as well as the accuracy of distributed optical fiber for rock fracture detection and discrimination. To this end, the research and development of a new system suitable for rock deformation observation are presented. The threshold value of the optical fiber test technology for rock fracture response and the time and space location of fracture generation are explored in the study. Therefore, the research content can improve the monitoring and early warning ability of rock mass stability of underground engineering and deepen our understanding of the mechanism of rock structure fracture.

## 2. The Basic Principle of BOTDR Optical Fiber Testing Technology

Since the 1980’s, optical fiber sensing technology has been widely used in aerospace, military, environmental and structural monitoring [34]. In the early days, the temperature-sensitive characteristics of optical fiber testing technology were mainly used to test the hydration heat of a concrete structure, water conservancy project leakage, and other related changes in the temperature field [35]. The BOTDR distributed optical fiber strain test has been successfully used in the deformation monitoring of large-scale foundation engineering, landslides, tunnels, foundation pits, and so on.

Brillouin scattering light is produced by the interaction of pump light and acoustic phonon, which belongs to one of the inelastic scatterings. The frequency shift of scattering light relative to the incident light is determined by the elastic mechanical properties and acoustic properties of the medium itself, as shown in Equation (1) [36]:

(1) $$v_{B}=\frac{2n}{\lambda }\sqrt{\frac{(1-\mu )E}{(1+\mu )(1-2\mu )\rho }}$$

where $v_{B}$ is the Brillouin frequency shift, n is the refractive index coefficient of optical fiber, λ is the wavelength of the incident light, E, μ and ρ are the Young’s modulus, Poisson’s ratio and density of the medium, respectively.

**Figure 1:**
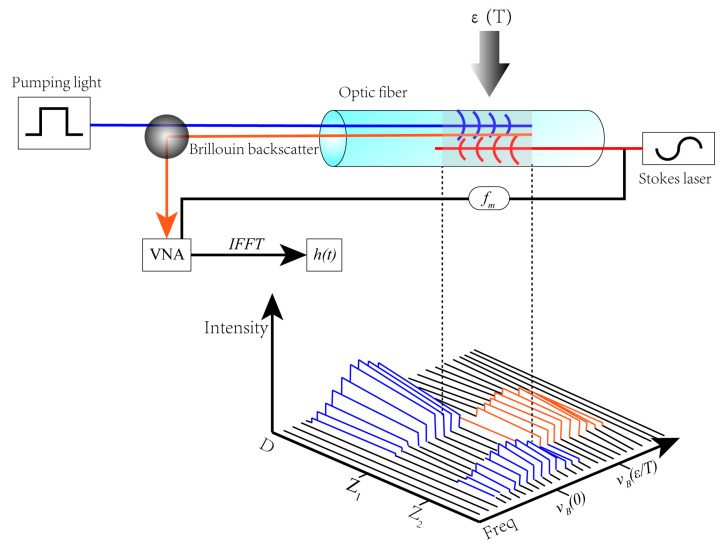
Principle of Brillouin optical time domain reflectometer (BOTDR) distributed optical fiber testing technology [38].

When the external strain or temperature changes, the Brillouin scattering wavelength will shift Figure 1 which can be expressed as Equation (2) [37]:

(2) $$v_{B}(\varepsilon ,T)=v_{B}(0)+\frac{\partial v_{B}(\varepsilon )}{\partial \varepsilon }\cdot \varepsilon +\frac{\partial v_{B}(T)}{\partial T}\cdot T$$

where ε, T are the strain and temperature of the test environment where the optical fiber is located; $v_{B}(\varepsilon ,T)$ is the Brillouin frequency shift; $v_{B}(0)$ is the Brillouin frequency shift in a free state of optical fiber; $\partial v_{B}(\varepsilon )/\partial \varepsilon$, $\partial v_{B}(T)/\partial T$ represent the strain coefficient and temperature coefficient, respectively.

Zhang D studied the temperature measurement using BOTDR and realized that when the change in the ambient temperature is small, the strain test can be conducted directly without considering the influence of temperature on wavelength. In this case, the test parameter expression can be expressed as Equation (3) [39]:

(3) $$v_{B}(\varepsilon )=v_{B}(0)+\frac{\partial v_{B}(\varepsilon )}{\partial \varepsilon }\cdot \varepsilon$$

The strain coefficient can be set as C, and its value is obtained by an indoor calibration test. Therefore, the test strain can be expressed as Equation (4) [40]:

(4) $$\varepsilon =\frac{v_{B}(\varepsilon )-v_{B}(0)}{C}$$

## 3. Experimental Process of Uniaxial Compression

### 3.1. Sample Preparation

Granite has a large compressive strength and can bear a larger axial force. To obtain more data in the test, the sample made of granite is suitable for this study. The granite samples from Wenchuan and Langfang in China are collected for the test. Following the standard recommended by the International Society for Rock Mechanics and Rock Engineering (ISRM) [16,41], the sample is processed into two groups of cylinder samples with a diameter of 50 mm and a length of 100 mm. The flatness, parallelism, and perpendicularity of the sample meet the specification requirements in terms of geometric accuracy. Table 1 display the main parameters of the sample.

### 3.2. Testing Instrument

**Figure 2:**
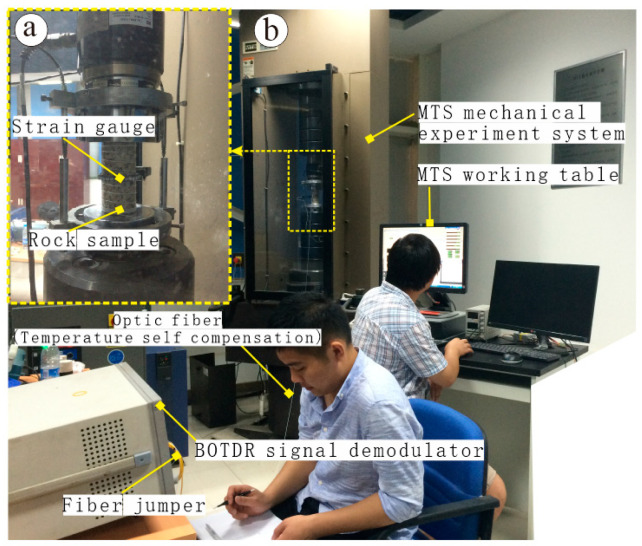
Test diagram of rock uniaxial fracturing system. (**a**) shows the strain gauge collection device; (**b**) shows the test system global diagram

The MTS testing system is the most advanced rock servo testing machine in the world, which is manufactured by the MTS Corporation of America. It is mainly used to test the mechanical properties and seepage characteristics of rock, concrete and coal mass under complex stress conditions. The measurement accuracy of MTS is high and stable, and the data acquisition at high and low speed can be controlled by force, displacement, axial strain, transverse strain, etc. In the test, the MTS-815 mechanical test system is used to compress the rock samples. The circular displacement and strain are obtained by using the strain track and the bonded BOTDR distributed optical fiber sensor, as shown in Figure 2. An Av6419 optical fiber strain distribution tester is used in the distributed optical fiber testing system, with pulse width of 10 ns, strain testing accuracy of ±40 με, sampling spatial resolution of 5 cm, frequency sampling range of 9.9~11.9 GHz, scanning interval of 5 MHz and an average number of times of 2^13^. BOTDR optical fiber test technology uses a single-end testing method, that is, the optical path does not need to form a loop. If the optical fiber breaks in the test, the test data from the test host to the breakpoint can still be obtained.

Considering the dual sensitivity of temperature and strain in BOTDR test technology, the temperature compensation adopts the form of self-compensation, that is to say, in the test, the optical fiber in the natural suspended state outside the winding lithological sample section is used as the test section for temperature compensation, and the frequency shift of the incident light in the natural state section is compared through multiple tests. If the indoor temperature has an impact on the test results, the interference caused by the temperature is put forward during data processing. If the indoor temperature does not affect the test results, it is not necessary to eliminate the data, and the strain data can be processed directly according to Equation (4).

### 3.3. Arrangement of Optical Fiber Sensors

The proper selection and arrangement of optical fiber sensors is fundamental in optical fiber testing technology. At present, a variety of optical fiber sensors have been developed for different test objects. The reasonable selection of sensors can provide better test data. As the diameter of the bare fiber is only 0.25 mm, it is easy damaged during inline layout and tests. In the test, single-mode tight sleeve optical fiber is selected, its model is G652b, and its core diameter is 0.9 mm. This kind of fiber sheath has a small elastic modulus and good strain transmission. During the test, it can not only protect the optical fiber, but also cooperate with the rock failure response to ensure the test accuracy. The refractive index of the selected core is 1.467.

**Figure 3:**
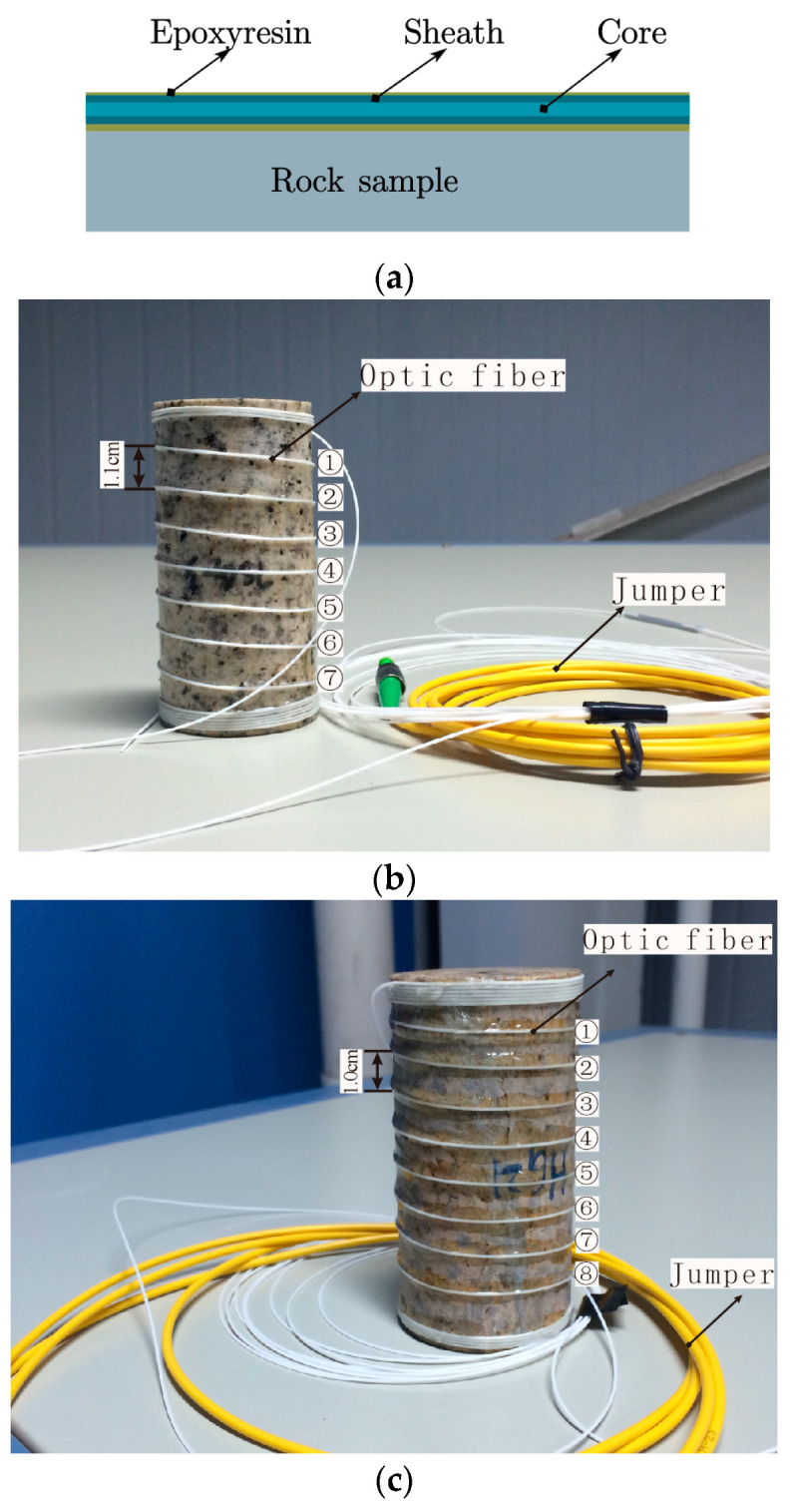
Layout description of optical fiber sensors; (**a**) shows the optical fiber pasting method; (**b**) and (**c**) show the optical fiber arrangement on the surface of sample No.0 and No.3.

The optical fiber is pasted on the surface of the rock sample in the form of bonding with the cement of the epoxy resin system. According to the research of related cementitious materials, it is shown that the physical properties of epoxy resin can realize the coupling between the optical fiber and rock sample, and the test will not affect the surface cracking of the sample [42]. Its cementation ability can make the optical fiber coordinate better with the specimen deformation. As shown in Figure 3, the spiral winding interval of No.0 rock sample optical fiber is 1.1 cm, and seven circles in the middle are selected as the data analysis section; the No.3 rock sample is also spirally wound with an interval of 1 cm. Eight circles in the middle are selected as the data analysis section. The length of the optical fiber on the rock surface is 17.1 cm, the total length of sample 0 is 119.7 cm, while the total length of sample 3 is 134.4 cm. To obtain relatively accurate test data, the length of the spiral winding in the middle of the sample is taken as the effective calculation length.

### 3.4. Arrangement of Optical Fiber Sensors

There are two experimental processes designed in this fracturing test. The first process is the uniaxial loading of the No.0 rock sample, which is the stage of elastic deformation, and the rock sample does not fracture. The stress–strain response of distributed fiber and MTS are compared to evaluate whether the distributed optical fiber testing technology has the feasibility of rock sample deformation observation. The second process involves loading the No.3 rock sample until the rock is fractured. A comparison of the stress–strain test results of the distributed optical fiber and MTS test system is explored in this process. At the same time, the distributed optical fiber test technology is also implemented to evaluate the recognition effect of the generation and evolution process of rock sample fracture. In the test, the rock samples are preloaded after the layout of the circumferential strain gauge is completed. The axial load applied by preloading is 2.5 KN. In these two processes, the axial continuous load is carried out according to the load gradient value of 0.5 KN/s. During the process, optical fiber data are collected in coordination. Among them, the first process stops after the total load reaches 200 KN, the rock sample is not fractured, and the process is incomplete, which is referred to as process A. In the early stage of this process, the optical fiber test data are collected for every 10 KN of load increase. Six groups of data are collected. Then, seven groups of data are collected for every 20 KN of load increase. A total of 13 groups of optical fiber test data are collected. In the second process, the optical fiber data is collected continuously according to the axial gradient value of 0.5 KN, and the optical fiber data collection is stopped until the rock sample breaks. In this process, the deformation and failure process of the rock sample is complete. When the rock sample is damaged, deformation occurs with a fracture sound, which is marked as process B. The background data and preloaded data for the optical fiber are collected before the test.

## 4. Test Results and Discussion

**Figure 4:**
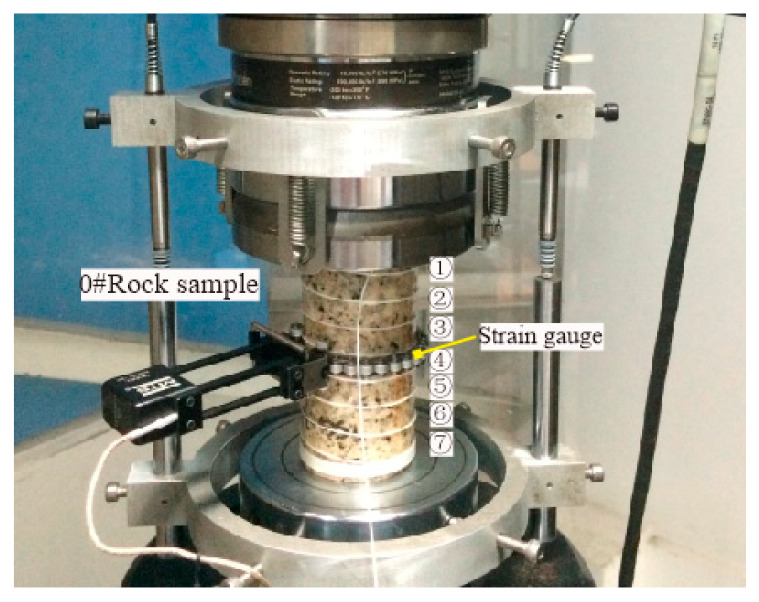
Layout position of the circumferential strain gauge.

MTS-815 rock mechanics servo equipment is widely recognized as having a good testing performance. Therefore, the test data obtained by the MTS test system is selected as the reference value. The distributed optical fiber is wound on the surface of the rock sample, and the circumferential strain gauge is arranged in the middle of the rock sample, which is offset from the position near the optical fiber winding, to obtain the ring displacement in the process of fracturing at this position. The layout is shown in Figure 4. Before the fracturing of the No.0 rock sample, the height position of the measuring circumferential strain gauge is measured, and the position of the rock sample at the height of the optical fiber measuring line is estimated, which locates at around 75 cm of the optical fiber data test section. Similarly, the No.3 rock sample uses the same method to locate the corresponding points of the optical fiber and circular extensometer, which are located at around 70 cm in the optical fiber test section. By comparing the strain trend of the adjacent points in the two processes, the test results of the BOTDR distributed optical fiber sensor are compared with those of MTS, and some preliminary results are obtained.

### 4.1. Analysis of The Deformation Process of the Rock Sample

**Figure 5:**
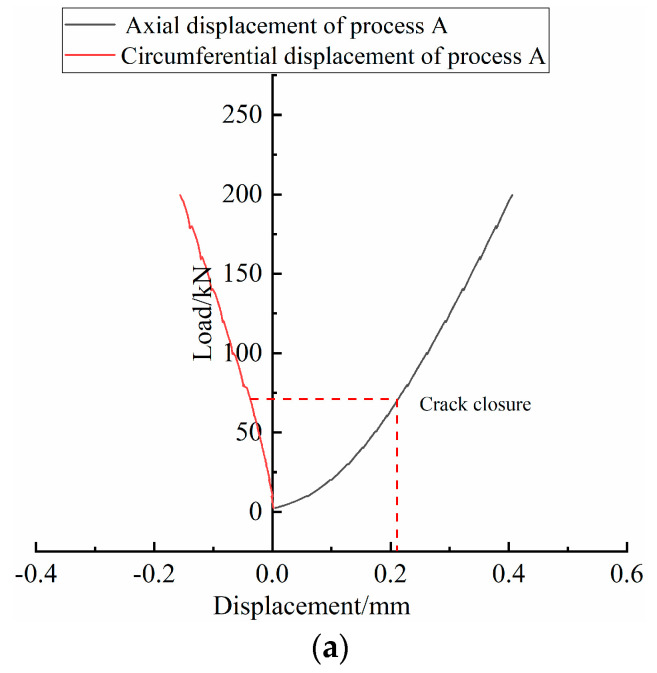

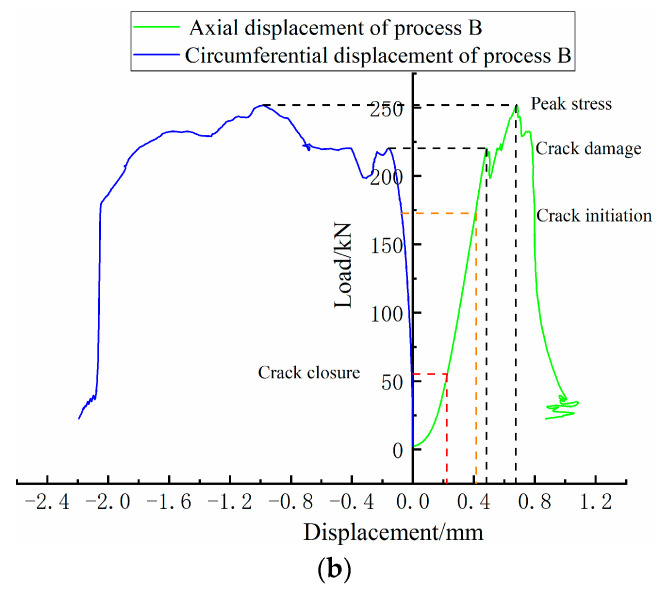
Displacement curve of rock sample during loading; (**a**) shows axial and circumferential displacement of the No.0 rock sample during loading; (**b**) shows axial and circumferential displacement of the No.3 rock sample during loading.

The rock sample in this test is granite, which has a relatively high compressive strength [43]. The two processes of A and B are designed in the test to provide better observations of the change in fracture surface strain under the uniaxial condition. Moreover, the axial displacement and the circumferential strain gauge displacement show an increasing trend with the increase in load. The changes are shown in Figure 5. By comparing the data, the circular displacement is smaller than the axial displacement. Due to the temporary suspension of the load applied by the MTS, a clear step-like shape can be observed, as shown in Figure 5a. The maximum axial load applied during process A is less than 200 kN, and the rock samples are not damaged. The load–displacement curve shows that the fracturing process is incomplete. In process B, the acquisitions of fiber optic data and rock fracturing are conducted simultaneously. During the process, the load increases continuously. The curve obtained by the MTS test system is relatively smooth, the data continuity is better, and the maximum peak axial stress of the load has reached 128.25 Mpa. From Figure 5b, the maximum axial load is 251.69 kN. It can be observed that the rock sample breaks when the load increases to 220.20 kN and the axial pressure decreases, but the deformation of the rock sample increases continuously. When the load increases to the peak value, the rock sample is destroyed and the final load decreases from the highest point to almost zero.

### 4.2. Optical Fiber Test Analysis of Process A

**Figure 6:**
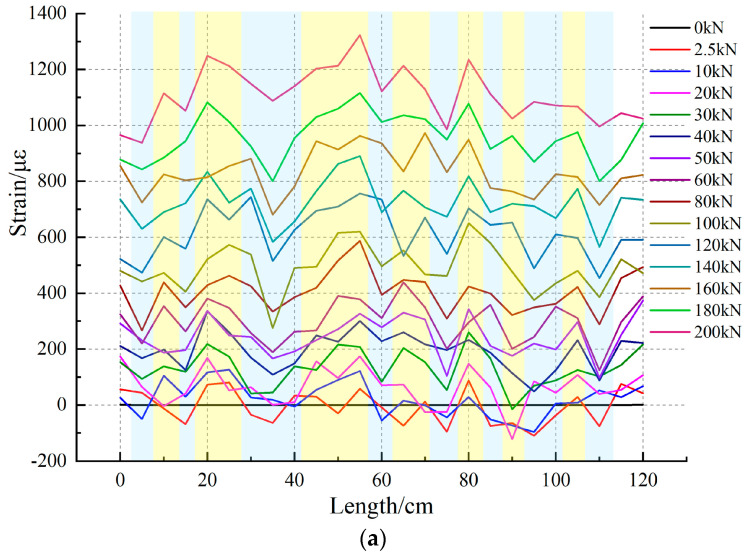

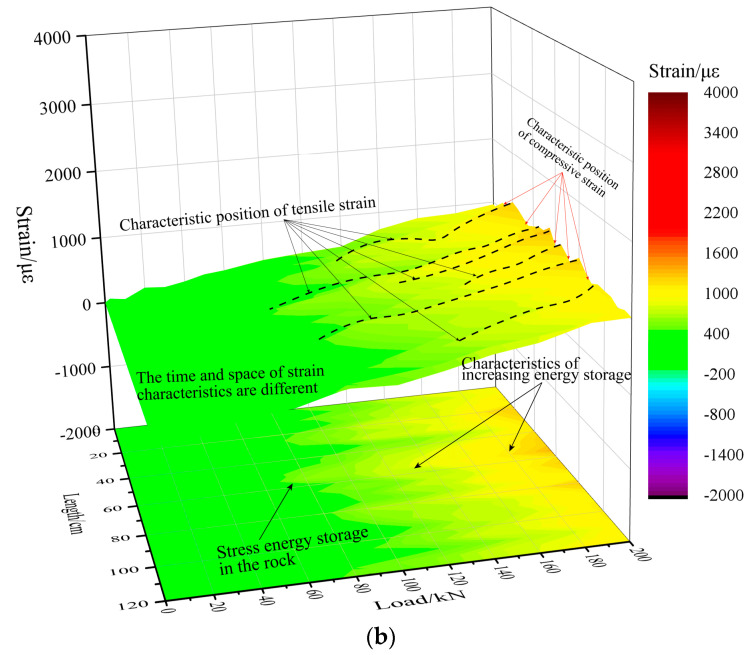
Distributed optical fiber test results of 0# rock sample surface strain; (**a**) shows the fiber optic strain for loading line length of No.0 rock sample; (**b**) shows the strain time–space nephogram of No.0 rock sample.

The strain distribution obtained under the uniaxial condition of No.0 rock sample is shown in Figure 6. The test values of rock samples before the load are selected as the initial data of the background, and the data collected during the process are inferior to the initial data. The strain values of No.0 rock samples wrapped around the optical fiber section under uniaxial compression are obtained. Since the ambient test temperature is constant at 21 ℃ and the temperature difference is less than 2 ℃, the ambient temperature has little effect on the data observation after the free-section optical fiber difference, so the influence of temperature on the test can be ignored [30,44].

The surface strain test data of the distributed optical fiber are shown in Figure 6a; it can be seen that the strain data of optical fiber in the test section increase gradually with the increase in load, and the increased area is the same as the test step. The test data also show that the optical fibers display different strain characteristics in the data analysis section. This indicates that, under load conditions, rock samples have different strain responses due to anisotropy. During the loading from 2.5 kN to 20 kN, the optical fiber strain data on the surface of rock samples show local compressive strain, i.e., strains less than zero appear at some locations. This may be caused by the closure of the internal pore, fissure structure, filling, and the internal structural surface of rock samples under a load, thus resulting in local volume compression. The corresponding characteristics are basically in agreement with the load–displacement curves. As the load increases, this volume compression tendency slows down and gradually turns into a volume increase. Similarly, the strain curve of the optical fiber test increases with the increase in load, and the strain values are different in the linear range, presenting non-linear characteristics.

The maximum local circumferential strain in process A reaches 1323 με when the load increases to 200 kN. Figure 6b presents the strain contour of rock sample No.0. The long section of the line that the volume changes near the middle and upper parts of the No.0 sample is larger than those at the lower part during uniaxial fracturing. This indicates that, under uniaxial load, the differences in the upper physical properties of the No.0 rock sample are weaker than those of the lower properties. This suggests, that if the rock is destroyed, deformation and destruction will take place preferentially around the marked points. At the same time, the strain distribution also reflects the fact that the tension failure and shear failure all exist during the failure process of rock mass, in which, while the tension failure is more obvious, the advantage of optical fiber line positioning can also be seen through the test results. By marking the indication points in Figure 6b, the deformation characteristics of the surface of rock sample No.0 can be observed, which are expressed by the time, space, and strain of the surface deformation. The test results are more intuitive and clearer.

**Figure 7:**
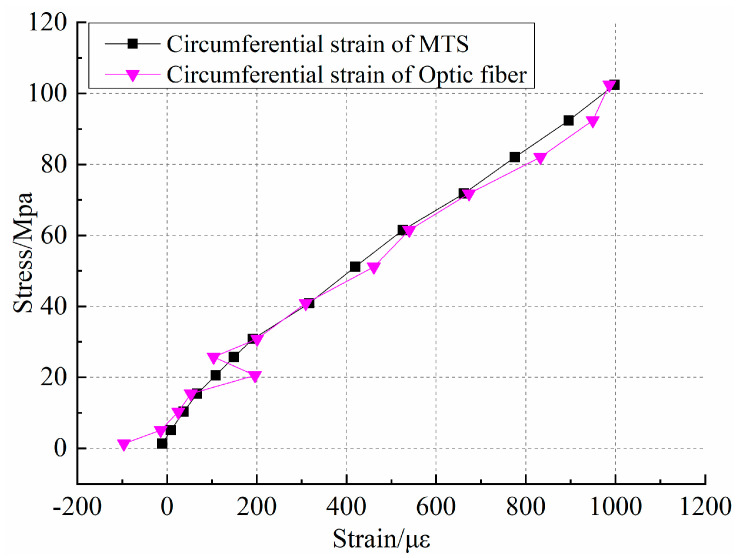
Comparison of circumferential strain between distributed optical fiber and MTS.

To evaluate the test results of the distributed optical fiber testing technology in terms of the circumferential strain, Figure 7 shows the comparison results of the MTS test circumferential strain and the optical fiber test circumferential strain. From the stress–strain curves, the strain curve shows an increasing trend with the increase in stress. By comparing the two test curves, the strain characteristics of distributed optical fibers are in good agreement with the toroidal strain characteristics of the MTS test. At the initial stage of fracturing under uniaxial loading conditions at an 0~25 Mpa interval, a significant strain jump can be observed, but the overall trend is still increasing, which indicates that, under axial load, the formation of cracks and the closure of internal pores during rock deformation produces a strong disturbance in the optical fiber. The optical fiber captures the change well during rock sample compaction. Based on the analysis of MTS test data, it can be seen that the curve is characterized by elastic deformation during the application of 25 Mpa to 100 Mpa axial stress, and the circumferential strain gauge shows a stable performance during this process. During this process, the measured results of the distributed optical fiber are slightly larger than those of the circumferential extensometer, which indicates that the optical fiber is more sensitive to the surface deformation of rock samples. Comparing the distributed optical fiber test results, the toroidal strain trend and measurement accuracy at the same position of the optical fiber are very close to those of the MTS test.

**Figure 8:**
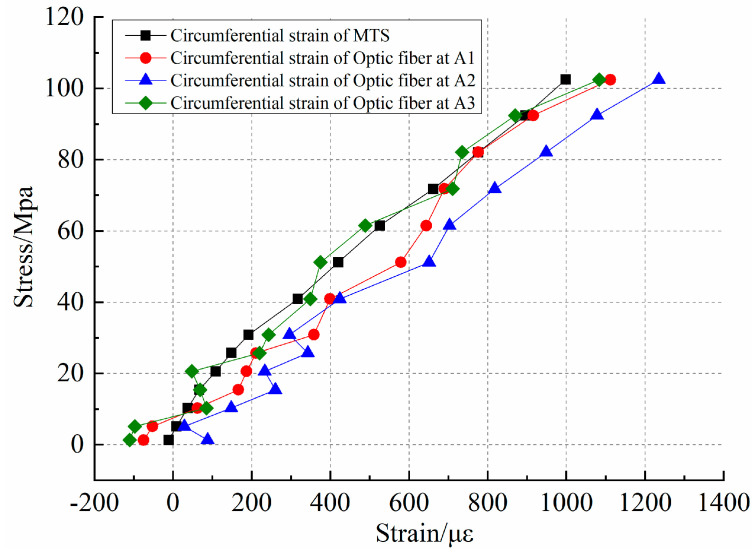
Stress–strain results of the adjacent points of the circumferential strain gauge.

Figure 8 shows the point taken at the optical fiber data section near the position of the circumferential strain gauge, where point A1 and point A2 are taken at the upper part of the circumferential strain gauge and point A3 at the lower part of the circumferential strain gauge. The distributed optical fiber can obtain the distribution characteristics of rock deformation and failure in the range of linear arrangement. Compared with the traditional point sensor, the distributed optical fiber has the feasibility to observe the fracture geometry information of the rock sample and can provide early warning information as a rock fracture precursor.

Therefore, the relative errors of the two test results of distributed optical fiber and MTS can be calculated as:

(5) $$E=\frac{\left|T_{x}-T_{x^{\prime }}\right|}{\left|T_{x^{\prime }}\right|}*100\%=\frac{\left|\Delta x\right|}{\left|T_{x^{\prime }}\right|}*\%$$

where E represents the relative error; $T_{x}$ is the optical fiber measurement value; $T_{x^{\prime }}$ represents the MTS measurement result.

The purpose of the process is to compare the feasibility of distributed optical fiber testing technology for circumferential strain testing. It can be seen from Figure 7 and Figure 8 that both the data trends are the same, but the curve characteristics are not exactly consistent. Therefore, an analysis of relative error at the same point height of the distributed optical fiber and circumferential extensometer is conducted. The results are shown in Table 2. The response error of the optical fiber test signal is large in the early stage. With the increase in load, the minimum test error of distributed optical fiber and MTS is 1.21%. In the early circumferential strain test, although the values are less than 100 με, the relative error will be very large once there is a deviation. In the later stage, the error decreases with the change in the reference value. At the same time, due to the early preloading process of the No.0 rock sample, the effect of optical fiber pasting, and the signal response to the rock sample in the compaction process, will have a compound impact on the data acquisition. In general, the distributed optical fiber shows good consistency with the MTS test results in the fracturing process. The distributed optical fiber testing technology provides the possibility for the observation of rock fracture evolution.

In the test of process, A, the distributed optical fiber testing technology tests the rock in a contact manner. Moreover, it responds well to the early deformation characteristics of the rocks. Compared with the 3D DIC test technology, the distributed optical fiber test technology can obtain the precursor response of rock cracks at an early stage, but it cannot provide the characteristics of image recording like 3D DIC [17]. However, the optical fiber testing technology will have less work to do in terms of data expression and sorting. In engineering applications, the coupling test can be conducted with the rock mass through the drilling form to obtain the corresponding test results. Nevertheless, this method is not as good as 3D DIC when it comes to avoiding the interference caused by rock structure and bedding [19]. To gain a deeper understanding of the distributed fiber’s response to rock failure, the process B tests were conducted.

### 4.3. Optical Fiber Test Analysis of Process B

**Figure 9:**
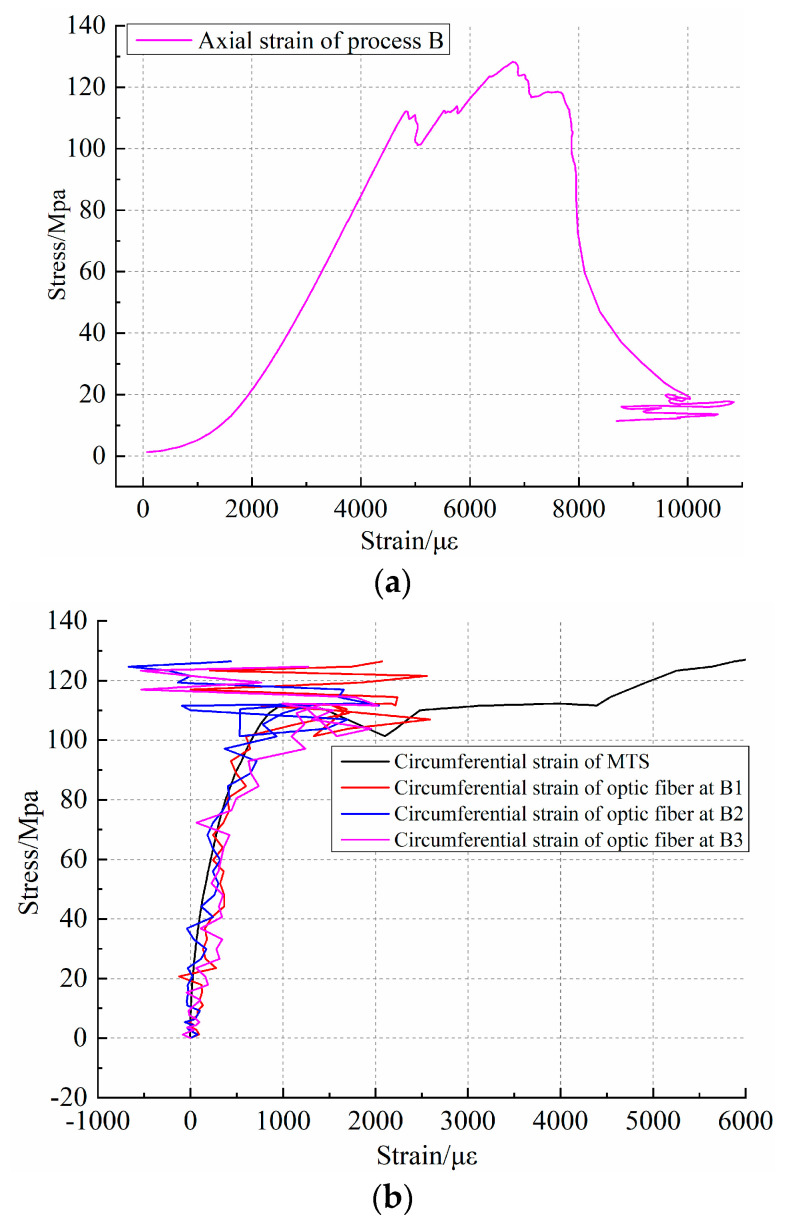
Fracture stress–strain results of No.3 rock sample; (**a**) shows axial stress–strain curve of No.3 rock sample under loading; (**b**) comparison of hoop strain measured by distributed optical fiber and MTS for No.3 rock sample.

To further verify the identification of rock fracture and its evolution process by distributed optical fiber testing technology, rock sample No.3 is loaded according to the design scheme of process B. In the test, the distributed optical fiber test data acquisition mode is continuous automatic acquisition [45]. During acquisition, the MTS test system load is applied in the manner described in Section 3.4 until the complete destruction of the rock sample, and data acquisition is stopped. The axial stress–strain curve of the 3# rock sample under load is shown in Figure 9a, and the trend of the curve is the same as that of the axial displacement. Therefore, referring to the ring strain comparison mode of the No.0 rock sample, the results of the MTS test system during the No.3 fracturing process are compared with those of the distributed optical fiber test system. The position of the ring displacement extensometer is taken separately, and the optical fiber test points B1, B2, and B3 are selected for the top, middle and bottom part. The data results of the two test methods are shown in Figure 9b. Figure 9a shows that the fracturing process of rock sample No.3 conforms to three stages of deformation under uniaxial compression: compaction process, elastic deformation, and plastic deformation. According to the curve, the first fracture occurs when the stress reaches 113.88 Mpa, but its overall stability is good, and the sample is not destroyed. With increasing load, the stress decreases after the final failure of 128.25 Mpa. Through the test curve shown in Figure 9b, it is found that the trend of the distributed optical fiber circumferential strain test results is similar to that of the MTS test system, but the data smoothness is poor and there are relatively large variances in the test results. In addition, there is a threshold at the end of the test. This indicates that the test results will change irregularly when the test strain exceeds the threshold value. According to the application research of distributed optical fiber testing by some scholars, it is shown that when optical fiber testing exceeds the threshold value and is not broken, the data of its measuring line segment can be obtained, but the test results are not accurate. The test results obtained in this way will not truly reflect the degree and characteristics of rock deformation. Therefore, in the process of No.3 rock sample fracturing, when the stress reaches 110.04 MPa, the optical fiber test results show an irregular response, and no longer give effective information. The data jump in the fracturing process of the No.3 rock sample is like that of the No.1 rock sample, which also reflects the development and expansion of micro-fractures in the fracturing process of a rock sample. Through two sets of rock fracturing tests, the load range of 100~110 Mpa can be set as the threshold value of this granite sample’s fracture. In this threshold range, the strain characterization of the fiber optic sensor is well matched with the MTS test results. However, if it goes beyond this range, the optical fiber test data will be inaccurate.

**Figure 10:**
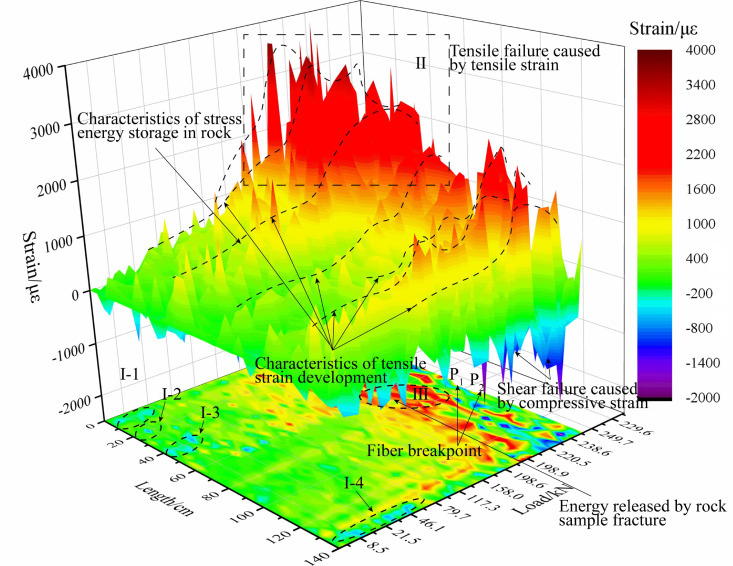
Strain test results of No.3 rock sample with distributed optical fiber.

Figure 10 shows the distributed fiber strain distribution cloud of process B. Through the strain distribution curve, the non-uniform deformation occurs under the load condition of the sample, with local volume expansion and local volume contraction on the surface of the sample. This deformation pattern indicates that the rock sample failure is induced by both the shear failure and tension failure between the structural planes. For example, in the process of 3000–4000 s load application, when the maximum load reaches 250.69 Mpa, the No.3 rock sample is completely damaged. During the process of fracturing, the optical fiber measuring line of 0–60 cm mainly shows tensile strain, while 108–140 cm shows large compressive strain at local points in the later stage of fracture. Like the early stage of fracture of the No.0 rock sample, they all show strain characteristics of tension and pressure coexistence. This shows that at the beginning of the load, the primary fracture of the closed rock sample is compacted, and with the continuous increase in the load, the test curve in the circumferential range not only mainly shows tensile strain, but also appears to show compressive strain at local points due to the existence of a weak structural plane. Moreover, during the loading process, the surface strain of the rock sample does not have the same tensile strain or compressive strain. For example, the distance between the two measuring lines is 60 cm. In this process, the transition from early compressive strain to tensile strain gradually occurs, such as I-1, I-2, I-3, I-4. The maximum compressive strain is −880 με, and the maximum tensile strain reaches 2620 με. It can also be seen from the distributed fiber strain distribution that the increase in tensile strain is not uniform, and there is a mutation in the local point or a change in the gradient band. Therefore, this sudden change and continuous change in gradient zone can be used to determine the time and distribution law of the rock surface deformation. Through the test, it can be found that, if fracture develops in the rock, the value change in the BOTDR distributed optical fiber test is usually greater than 600~800 με. Consequently, in combination with the research conclusions and the results of this test, the characteristics of optical fiber strain response for rock mass fracture can be concluded as follows: when the strain at a point increases continuously and the value increases greatly or, after a sudden increase, then falls at a certain point, the fracture development on the rock surface can be inferred. In the fracturing test of process B rock samples, the maximum tensile strain of the optical fiber test reaches 3860 με, which is distributed in zone II of Figure 10. In the final fracturing process, the fracturing of No.3 rock sample penetrates through the sample and releases huge energy, which not only makes a loud noise, but also causes the breakage of the winding optical fiber. During the fracturing process of the No.3 rock sample, it can be determined by the test results that the fissure expansion of the rock first occurs when the loading time reaches 3024 s, and this is determined as the P1 points of area III in Figure 10. At 3744s, the cracks penetrate the rock sample and cause the optical fiber to break, corresponding to the P2 points. The breaking time corresponds clearly with the MTS system test system in terms of the load relationship. Fiber optic data are continuously collected during the fracturing process of the No.3 rock sample, 63 sets of data are collected. Strain data within the length of the measuring line can reflect obvious changes in a rock sample in time and space and can form an early strain expression, which can provide a reference for the prediction of deformation and the failure of rock.

**Figure 11:**
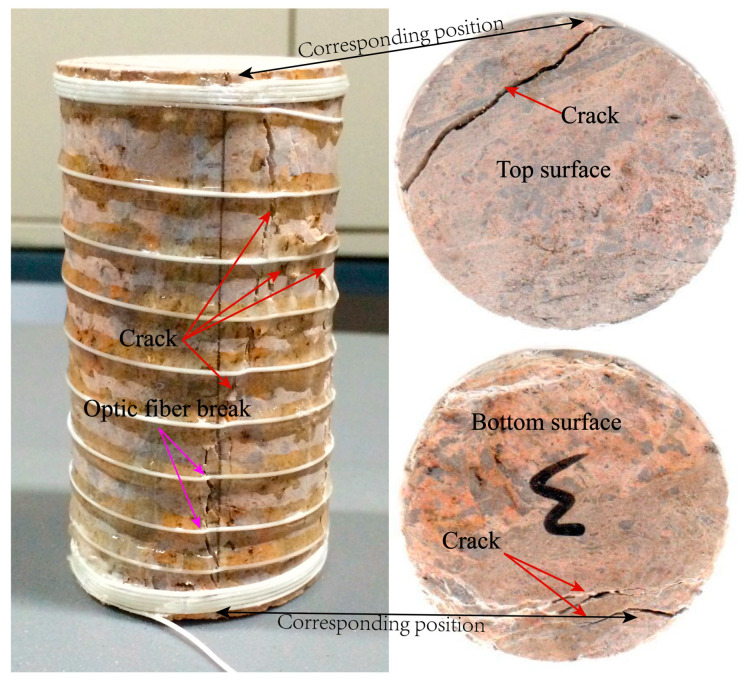
Fracture surface damage characteristics of No.3 rock sample.

The fracturing process of the No.3 rock sample is complete under uniaxial conditions. After the load is applied, its surface morphology is recorded, as shown in Figure 11. The figure shows the fracture morphology of the side, top, and bottom of the rock sample after fracturing. By comparing the projection of strain on the bottom, the areas of surface tension failure and shear failure can be delineated. The point marked with a serial number is the position where there is a strain change feature; the change feature can locate the fracture range in the early stage, as shown in area II in Figure 10. The strain area corresponds to the fracture surface failure area of the No.3 rock sample and they show good agreement. However, in this test, the fracture morphology and structure of the No.3 rock sample in the later stage of fracturing fail to achieve quantitative evaluation. Therefore, the test results can show that the reasonable arrangement of distributed optical fiber can be used to capture the precursory information of rock mass macro fractures and to improve the ability of disaster prevention and early warning in the engineering field.

## 5. Conclusions

The research results of this experiment are summarized as follows:

(1) Distributed optical fiber is used to observe strain during the rock fracturing process under uniaxial compression. The optical fiber testing technology has good consistency with the MTS test results in terms of its circumferential strain response. During the testing process, the minimum relative error of local points in the circumferential position reaches 1.27%. BOTDR distributed optical fiber technology introduces a new testing method for rock deformation and failure observation and provides basic data for underground engineering applications.
(2) Distributed optical fiber testing has a threshold value for rock fracture observation. Generally, rock failure occurs when a tensile strain reaches 600~800 με, which can be better reflected in the early and middle stages of the testing process. The process of micro-deformation of rock and surface strain before failure can be captured, and thus the precursor information of rock failure can be distinguished, and the disaster prevention and warning ability of the project can be improved. The test results of the No.0 and No.3 rock samples also indicate that the thresholds for optical fiber testing are different for different rocks.
(3) Distributed optical fiber testing technology can identify the time of rock fracture development, reflect the tension and shear damage zone of surface strain before rock sample fracture, and delineate the location of failure in microscopical view. In this experiment, the morphological imaging of tissue is incomplete. In practical engineering applications, the space and time scales of rock and soil deformation are larger than those of the test. A reasonable scheme design and optical fiber arrangement are helpful for distributed optical fiber testing technology to capture the process of rock mass deformation and destruction and to analyze the evolution law of rock mass.
(4) In this study, two groups of rock samples are used for fracturing tests; the comparison shows that the collection method of distributed optical fiber data, the mechanical properties of optical fiber, and the consolidation of the cementing agent will all have an impact on the test data. Improving the optical fiber performance and coupling treatment between the optical fiber and rock mass are key and these are difficult points that must be explored in the rock mass monitoring of distributed optical fiber testing technology in the future.

## Figures and Tables

**Table 1:** Parameters of the Sample.

Number	Collection Location	Diameter/mm	Length/mm
0#	Wenchuan	49.83	100.14
3#	Langfang	49.75	100.23

**Table 2:** Comparison of Process A Distributed Optical Fiber and MTS Test Results.

Load/kN	$T_{x}$/με	$T_{x^{\prime }}$/με	Δx	E/%
2.5	−96	−10.9	−85.1	783.17
10	−14	8.6	−22.6	262.19
20	25	36.7	−11.7	31.96
30	53	66.8	−13.8	20.71
40	196	108.7	87.3	80.36
50	104	149	−45	30.18
60	201	191.9	9.1	4.74
80	309	317.1	−8.1	2.55
100	461	419.7	41.3	9.84
120	540	525.7	14.3	2.73
140	673	661.8	11.2	1.69
160	832	775.9	56.1	7.23
180	949	895.8	53.2	5.94
200	986	998	−12	1.21

## References

[1] Deere D.U. (1964). Technical description of rock cores for engineering purposes. Rock Mech. Eng. Geol..

[2] Priest S.D., Hudson J.A. (1976). Discontinuity spacings in rock. Int. J. Rock Mech. Min. Sci. Geomech. Abstr..

[3] Dershowitz W.S., Einstein H.H. (1988). Characterizing rock joint geometry with joint system model. Rock Mech. Rock Eng..

[4] Huang R.Q., Dong X.J. (2008). Application of three-dimensional laser scanning and surveying it geological investigation of high rock slope. J. China Univ. Geosci..

[5] Zhou W., Shi X., Lu X., Qi C., Luan B., Liu F. (2020). The mechanical and microstructural properties of refuse mudstone-GGBS-red mud based geopolymer composites made with sand. Constr. Build. Mater..

[6] Dey T.N., Wang C.Y. (1981). Some mechanisms of microcrack growth and Interaction in compressive rock failure. Int. J. Rock Mech. Min. Sci. Geomech. Abstr..

[7] Bobet A. (2000). Modelling of crack initiation, propagation and coalescence in uniaxial compression. Rock Mech. Rock Eng..

[8] Knill J.L., Franklin J.A., Malone A.W. (1968). A study of acoustic emission from stressed rock. Int. J. Rock Mech. Min. Sci..

[9] Zhao Y., Huang J., Wang R. (1993). Real-time SEM observations of the microfracturing process in rock during a compression test. Int. J. Rock Mech. Min. Sci. Geomech..

[10] Zhao Y., Huang J., Wang R. (1992). Real-time observation of microfracturing process in rock during compression test. Chin. J. Rock Mech. Eng..

[11] Liu D.M., Cai M.F., Zhou Y.B., Chen Z.Y. (2006). Dynamic monitoring on developing process of rock cracks. Chin. J. Rock Mech. Eng..

[12] Kawakata H., Cho A., Yanagidani T., Shimada M. (1997). The observations of faulting in Westerly granite under triaxial compression by X Tay CT scan. Int. J Rock Mech. Min. Si..

[13] Ge X.R., Ren J.X., Pu Y., Ma W., Zhu Y. (1999). A real-in-time CT triaxial testing study of meso-damage evolution law of coal. Chin. J. Rock Mech. Eng..

[14] Wei S.M., Chai J. (2010). Strain transfer analysis of rock deformation based on FBG Sensing. J. Exp. Mech..

[15] Cheng-Kai F., Yan-Kun S., Qi L., Hai-Feng L., Zhi-Yong N., Xia-Ying L. (2017). Testing Technology of fiber Bragg grating in the shale damage experiments under uniaxial compression conditions. Rock Soil Mech..

[16] Munoz H., Taheri A., Chanda E.K. (2016). Pre-Peak and Post-Peak Rock Strain Characteristics During Uniaxial Compression by 3D Digital Image Correlation. Rock Mech. Rock Eng..

[17] Munoz H., Taheri A. (2017). Local Damage and Progressive Localization in Porous Sandstone During Cyclic Loading. Rock Mech. Rock Eng..

[18] Munoz H., Taheri A. (2020). Specimen aspect ratio and progressive field strain development of sandstone under uniaxial compression by three-dimensional digital image correlation. J. Rock Mech. Geotech. Eng..

[19] Munoz H., Taheri A., Chanda E.K. (2016). Fracture Energy-Based Brittleness Index Development and Brittleness Quantification by Pre-peak Strength Parameters in Rock Uniaxial Compression. Rock Mech. Rock Eng..

[20] Taheri A., Zhang Y., Munoz H. (2020). Performance of rock crack stress thresholds determination criteria and investigating strength and confining pressure effects. Constr. Build Mater..

[21] He T.M., Liu Z.Y., Li S.Y. (2015). Experimental Studies of Ultrasonic Tomography on Fangshan Granite Under Uniaxial Compression. CT Theory Appl..

[22] Yong-Shang M.A., Wei-Zhong C.H.E.N., Dian-Sen Y., Jian-Ping Y., Zhe G. (2017). Experimental study of brittle rock failure based on three-dimensional digital image correlation technique. Rock Soil Mech..

[23] Zhang Y.B., Wu W.R., Yao X.L., Liang P., Tian B.Z., Huang Y.L., Liang J.L. (2020). Acoustic emission-infrared characteristics and damage evolution of granite under uniaxial compression. Rock Soil Mech..

[24] Wu Z.S., Zhang H., Yang C.Q. (2010). Development and performance evaluation of no-slippage optical fiber as Brillouin scattering based distributed sensors. Struct. Health Monit..

[25] Shi B., Xu H.Z., Zhang D., Ding Y., Cui H.L., Gao J.Q., Chen B. A Study on BOTDR application in monitoring deformation of a tunnel. Proceedings of the First International Conference on Structural Health Monitoring and Intelligent Infrastructure.

[26] Liu J., Shi B., Zhang D., Sui H.B., Suo W.B. (2006). The experimental study of foundation pit deformation distribution based on BOTDR. Rock Soil Mech..

[27] Mendez A., Morse T.F. Applications of embedded optical fiber sensors in reinforced concrete buildings and structures. Proceedings of the SPIE-The International Society for Optical.

[28] Fuhr P.L., Huston D.R., Nelson M., Nelson O., Hu J., Mowat E., Spammer S., Tamm W. (1999). Fiber optic sensing of a bridge in Waterbury. Intell. Mater. Syst. Struct..

[29] Inaudi D., del Grosso A.E., Lanata F. (2001). Analysis of long-term deformation data from the San Giorgio harbor pier in Genoa. SPIE Proc..

[30] Zhang D., Zhang P.S., Shi B., Wang H.X., Li C.S. (2015). Monitoring and analysis of overburden deformation and failure using distributed fiber’ optic sensing. Chin. J. Geotech. Eng..

[31] Zhang P.S., Xu S.A., Guo L.Q., Wu R.X. (2020). Prospect and progress of deformation and failure monitoring technology of surrounding rock in stope. Coal Sci. Technol..

[32] Wei S.M., Chai J. (2008). Study of application of optical fiber Bragg grating sensing to uniaxial compression experiments of rock. Rock Soil Mech..

[33] Chai J., Du W., Yuan Q., Zhang D. (2019). Analysis of test method for physical model test of mining based on optical fiber sensing technology detection. Opt. Fiber Technol..

[34] Bao X., Zhou D.P., Baker C., Chen L. (2017). Recent development in the distributed fiber optic acoustic and ultrasonic detection. J. Lightwave Technol..

[35] Lanciano C., Salvini R. (2020). Monitoring of Strain and Temperature in an Open Pit Using Brillouin Distributed Optical Fiber Sensors. Sensors.

[36] Zhang D., Shi B., Cui H.L., Xu H.Z. (2004). Improvement of spatial resolution of Brillouin optical time domain reflectometer using spectral decomposition. Opt. Appl..

[37] Zhang D., Shi B., Wu Z.S., Shen S., Zhang Y. (2003). Distributed optical fiber sensor based on BOTDR and its application to structural health monitoring. China Civ. Eng. J..

[38] Wang X., Shi B., Wei G.Q., Cheng G., Zgang C. (2015). A Novel Technique for Civil and Geotechnical Engineering Monitoring: Performance and Characteristics of BOFDA. J. Dis. Prev. Mitig. Eng..

[39] Dan Z., Bin S.H.I., Hong-Zhong X., Junqi G., Hong Z. (2004). Experimental study on the deformation monitoring of reinforced concrete T-beam using BOTDR. J. Southeast Univ. (Nat. Sci. Ed.).

[40] Horiguchi T., Kurashima T., Tateda M. (1989). Tensile strain dependence of Brillouin frequency shift in silica optical fibers. IEEE Photonics Technol. Lett..

[41] Fairhurst C.E., Hudson J.A. (1999). Draft ISRM suggested method for the complete stress-strain curve for intact rock in uniaxial compression. Int. J. Rock Mech. Min. Sci..

[42] Sun Y., Li Q., Yang D., Fan C., Sun A. (2016). Investigation of the dynamic strain responses of sandstone using multichannel fiber-optic sensor arrays. Eng. Geol..

[43] Zhang Y., Zhang Z., Xue S., Wang R., Xiao M. (2020). Stability analysis of a typical landslide mass in the Three Gorges Reservoir under varying reservoir water levels. Environ. Earth Sci..

[44] Zhu H.H., Shi B., Yan J.F., Zhang J., Wang J. (2015). Investigation of the evolutionary process of a reinforced model slope using a fiber-optic monitoring network. Eng. Geol..

[45] Zhang L.F., Yang D.X., Chen Z.H., Liu A.C. (2020). Deformation and failure characteristics of sandstone under uniaxial compression using distributed fiber optic strain sensing. J. Rock Mech. Geotech. Eng..

